# Two open states of P2X receptor channels

**DOI:** 10.3389/fncel.2013.00215

**Published:** 2013-11-14

**Authors:** Milos B. Rokic, Stanko S. Stojilkovic

**Affiliations:** Section on Cellular Signaling, Program in Developmental Neuroscience, The Eunice Kennedy Shiver National Institute of Child Health and Human Development, National Institutes of HealthBethesda, MD, USA

**Keywords:** ATP, purinergic receptor channels, gating, pore opening, pore dilation, NMDG, YO-PRO-1

## Abstract

The occupancy of the orthosteric ligand binding sites of P2X receptor (P2XR) channels causes the rapid opening of a small cation-permeable pore, followed by a gradual dilation that renders the pore permeable to large organic cations. Electrophysiologically, this phenomenon was shown using whole-cell current recording on P2X2R-, P2X2/X5R-, P2X4R- and P2X7R-expressing cells that were bathed in N-methyl-D-glucamine (NMDG^+^)-containing buffers in the presence and/or absence of small monovalent and divalent cations. The pore dilation of P2X4R and P2X7R caused a secondary current growth, whereas that of P2X2R showed a sustained kinetic coupling of dilation and desensitization, leading to receptor channel closure. The pore size of the P2X7R open and dilated states was estimated to be approximately 0.85 nm and greater than 1 nm, respectively. The P2XR pore dilation was also observed in intact cells by measurement of fluorescent dye uptake/release, application of polyethylene glycols of different sizes, and atomic force microscopy. However, pore dilation was not observed at the single channel level. Structural data describing the dilated state are not available, and the relevance of orthosteric and allosteric ligand interactions to pore dilation was not studied.

## Introduction

P2X receptors (P2XRs) are adenosine triphosphate (ATP)-gated ion channels expressed in numerous excitable and non-excitable cells from various species, including humans (hP2XRs), rats (rP2XRs), mice (mP2XRs), and zebrafish (zP2X4.1R), where they contribute to the control of many physiological functions (Chizh and Illes, 2001; Vassort, 2001; North, 2002; Burnstock and Knight, 2004; Stojilkovic, 2009; Khakh and North, 2012). Mammalian P2X subunits exist in seven isoforms with the ability to form homo- and heterotrimers* in vitro* and in native tissues (Nicke et al., 1998; Kaczmarek-Hajek et al., 2012). The structure of zP2X4.1R in the closed state revealed that the core domains of the three subunits mutually intertwine, forming a central vertical cavity. The ectodomain is projected 0.7 nm above the plasma membrane and contains three vestibules along its central axis (Kawate et al., 2009). The structure of zP2X4.1R in the open state showed that the key residues for ATP binding are located approximately 0.45 nm from the plasma membrane (Hattori and Gouaux, 2012). Consistent with mutagenesis studies (Ennion et al., 2000; Jiang et al., 2000; Roberts and Evans, 2006; Yan et al., 2006; Zemkova et al., 2007), this structure revealed that the residues K67, K69, N293, R295, and K313 (rP2X4R numbering) are crucial for the recognition of phosphate groups, while the adenine moiety lies deeper in the binding pocket and is stabilized by hydrogen bonding (Hattori and Gouaux, 2012).

When bathed in physiological ion conditions, P2XRs respond to ATP stimulation with amplitude-modulated inward currents (North, 2002). Using whole-cell recording, these currents can be described by four parameters: (i) the activation time; (ii) the peak current amplitude; (iii) the rate of decay of the current amplitude during a sustained receptor stimulation; and (iv) the rate of decay of the current amplitude during agonist washout (Coddou et al., 2011). The activation time decreases and the peak current amplitude and rate of receptor desensitization increase with elevation in agonist concentration, whereas the rate of current deactivation is independent of agonist concentration; this phenomenon is well documented for rP2X4R using an ultra-rapid perfusion system (Yan et al., 2006). At the single channel level, activation is defined as the transition from the closed state to the open state, desensitization as the transition from the open state to the closed-desensitized state, and deactivation as the transition from the open state to the closed state (Egan et al., 2006). The existence of naïve (not previously exposed to ATP) and experienced (previously exposed to ATP) receptor states has also been proposed, which may reflect the phosphorylation state of P2XRs or other mechanisms accounting for short-term (less than 30 min) memory (Yan et al., 2010).

P2XRs are permeable to Na^+^, K^+^, and Ca^2+^ (Ding and Sachs, 1999), but the permeability to Ca^2+^ varies widely depending on the isoform (Evans et al., 1996; Soto et al., 1996; Virginio et al., 1998; Egan and Khakh, 2004). Some homomeric and heteromeric P2XRs also display a time-dependent modulation of ion selectivity by developing a new open state that permits relatively large cations to traverse the pore of the channel (Khakh et al., 1999; Virginio et al., 1999b). This phenomenon is called pore dilation, with the dilated pore representing a second open state with higher ion conductance. Our review focuses on the biophysical and biochemical aspects of pore dilation and transition to closed states, noting the isoform specificities.

## Historical perspectives

Extracellular ATP was initially suggested to affect permeabilization of the plasma membrane by two mechanisms: through its interaction with P2 receptors and by using a P2-independent mechanism to make pores large enough to allow for the permeation of substances with molecular weights (MW) ranging between 300 and 900 (Cockcroft and Gomperts, 1979; Steinberg et al., 1987; Tatham and Lindau, 1990). Other authors suggested that both actions were mediated by P2 receptors and introduced the term “P2Z” to describe a putative permeabilizing P2 receptor that required for activation high concentrations of ATP, lower concentrations of Benzoylbenzoyl adenosine- 5'-triphosphate (BzATP), and the removal of Ca^2+^/Mg^2+^ from the bath medium (Gordon, 1986). A receptor with a pharmacological profile typical of the P2Z receptor was cloned in 1996 and named P2X7R (Surprenant et al., 1996) and was able to permeabilize membranes (Virginio et al., 1999a). We now know that the rP2X7R transition from open to dilated states accounts for the permeabilizing action of ATP (Yan et al., 2008) and that the allosteric nature of Ca^2+^-dependent inhibition accounts for the stimulatory effects of divalent cation removal on ATP’s potency for activation (Yan et al., 2011). Initial experiments with rP2X2R (Virginio et al., 1999b) and rP2X4R (Khakh et al., 1999) have shown that the transition from the open to dilated state is not a unique characteristic of P2X7R. Experiments with other channels also revealed that pore dilation is not a P2XR-specific phenomenon (Khakh and Lester, 1999; Chung et al., 2008).

## Methods for studying pore dilation

In whole-cell recording, large organic cations such as *N-methyl-D-glucamine (NMDG*^+^; MW 195) are commonly used to evaluate changes in the permeability of P2XR pores during sustained receptor activation. NMDG^+^ is used alone or as a substitute for bath Na^+^ in the presence of other inorganic cations. Prior to ATP application, cells expressing P2XR are impermeable to NMDG^+^. The uptake of nucleic acid-binding dyes, including YO-PRO-1 and TO-PRO-1, ethidium bromide (MW 394), and propidium iodide (MW 668), is also a standard tool for measuring cell permeabilization. YO-PRO-1 has been shown to permeate through rP2X2R, rP2X4R, rP2X7R, mP2X7R, and hP2X7R (Khakh et al., 1999; Virginio et al., 1999a; Hibell et al., 2000; Chessell et al., 2001; Yan et al., 2008). Ethidium bromide, most commonly used in the visualization of DNA and RNA in electrophoresis gels, was used to show changes in the permeability of human and mouse P2X7R (Stokes et al., 2010; Tran et al., 2010). This application is also the case with propidium iodide (Sun et al., 2010) and TO-PRO-1 (Mankus et al., 2011). The uptake of Lucifer Yellow (MW 457) was used to study the permeation of native mP2X2R and mP2X7R in taste bud cells (Hayato et al., 2007). Cellular leakage of the Ca^2+^ probes Fura-2 (MW ranging between 636 and 1001) and Fura-FF (MW ranging between 658 and 1023) has provided further useful information about the dilation of rP2X2R and rP2X7R (Yan et al., 2008; Khadra et al., 2012). In addition, the size of the dilated pore was measured by the application of differently sized polyethylene glycols; those having a MW greater than or equal to 5000 blocked the increase in cation permeability in cells expressing rP2X7R, suggesting that the dilated pore is greater than 1 nm in diameter (Virginio et al., 1999a). Finally, rP2X4R (expressed in human 1321N1 cells) pore dilation was observed by atomic force microscopy (Shinozaki et al., 2009).

## Pore dilation accounts for biphasic currents

The current generated by naïve rP2X7R expressed in Human Embryionic Kidney 293 (HEK293) cells (Roger et al., 2008; Yan et al., 2008) and by hP2X7R expressed in *Xenopus* oocytes (Klapperstuck et al., 2001) features a biphasic response in whole-cell recordings; the initial rapid rise in current (I_1_) is accompanied by a secondary slow current growth (I_2_), the rate of which increases with agonist concentration (Yan et al., 2010). The I_2_ is also evident when intracellular Ca^2+^ measurements are used to indicate the receptor activity in both amphotericin-perforated and intact cells (Yan et al., 2010). This phenomenon suggests that neither the expression system nor the washout of secondary messengers or intracellular ions accounts for this P2X7R behavior.

**Figure 1:**
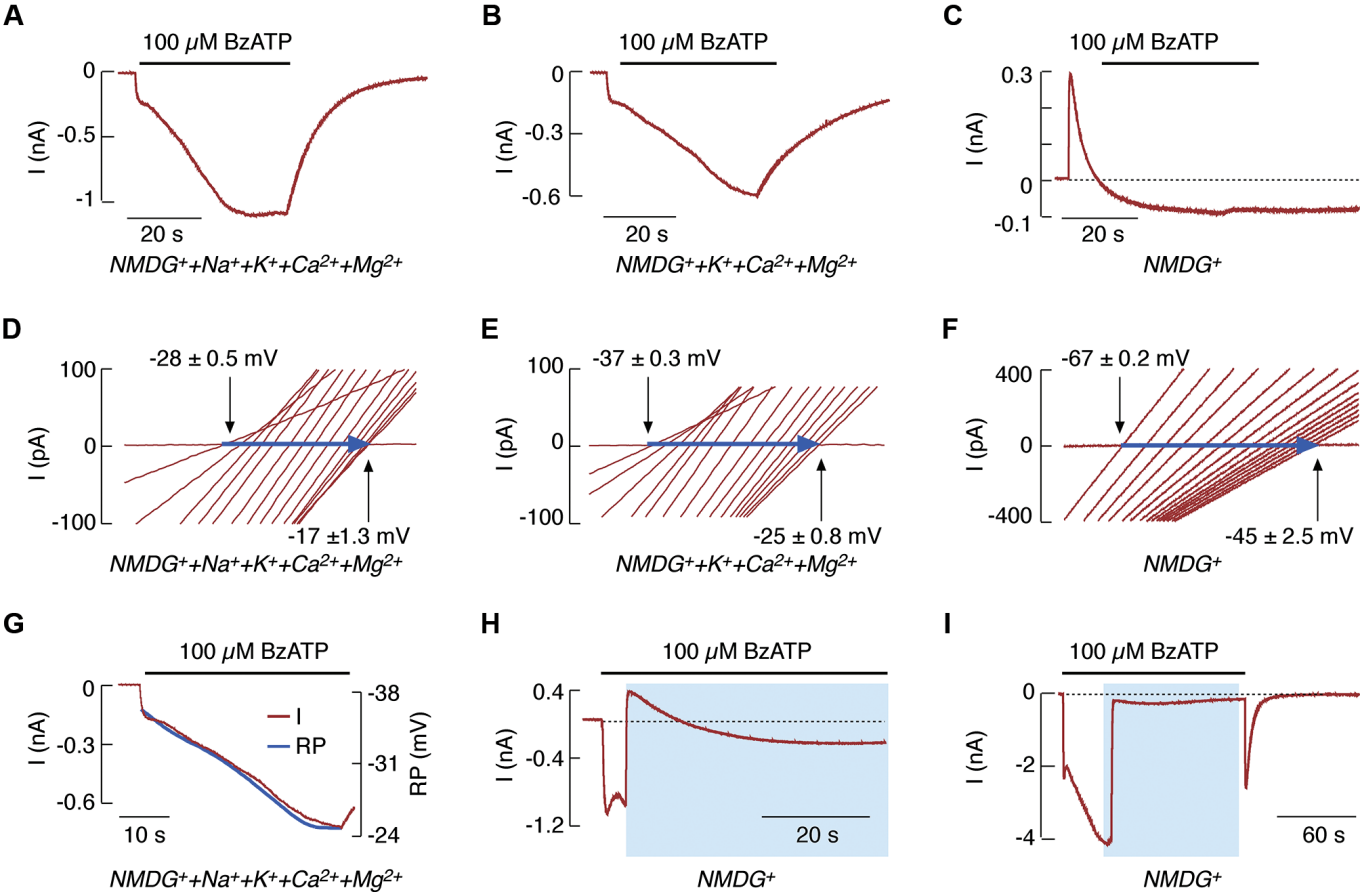
**NMDG^+^ permeability of rP2X7R expressed in HEK293 cells**. **(A–F)** Patterns of BzATP-induced currents **(A–C)** and reversal potentials **(D–F)** in cells continuously bathed in media containing NMDG^+^, Na^+^, K^+^, and divalent cations (**A** and **D**); NMDG^+^, K^+^, and divalent cations (**B** and **E**); and NMDG^+^ only (**C** and **F**). This figure (as well as the one following) shows that whole-cell current recordings were performed at a holding potential of −60 mV, and agonist was applied for 40 s. Voltage ramps were delivered twice per second during the 40 s agonist application to record positive shifts in reversal potential (horizontal blue arrows); only 15 traces (out of a total of 100) for the current-voltage relationship with equal time intervals are shown. **(G)** Comparison of the kinetics of secondary current growth (red trace) and of changes in reversal potential (RP; blue trace) in cells bathed in media containing NMDG^+^ and divalent cations during the 40 s application of BzATP. **(H and I)** 100 μM BzATP-induced current profiles in cells bathed in a normal physiological solution for 4 s **(H)** and 40 s **(I)**, and in NMDG^+^-containing media for the rest of the agonist application time. Horizontal black lines above the traces indicate the duration of BzATP application, and blue areas indicate the duration of NMDG^+^ application. Derived from Yan et al. (2008, 2010).

Substituting bath Na^+^ with NMDG^+^ permits current recordings in cells clamped at −60 mV and the detection of changes in reversal potential, as estimated by repetitive voltage-ramp pulses from −80 mV to +80 mV. This finding was used to support the hypothesis of rP2X7R pore dilation (Yan et al., 2008). The substitution of 90% (Figure 1A) and 100% (Figure 1B) of the extracellular Na^+^ with NMDG^+^ does not alter the biphasic response pattern of rP2X7R generated by the prolonged application of BzATP when Ca^2+^, Mg^2+^, and K^+^ are all present in the bath medium. Under repetitive voltage-ramp pulses from −80 to +80 mV, a positive shift was also found in the reversal potential (indicated by horizontal arrows in Figures 1D, E). The rate of reversal potential shift was highly comparable to the rate of I_2_ current growth (Figure 1G).

However, the time-course of rP2X7R agonist induced current was substantially different in cells bathed in NMDG^+^-containing medium lacking other cations (Figure 1C). The time-course consisted of an initial outward current, reflecting the movement of intracellular Na^+^ through the channel pore, followed by a shift to an inward current, reflecting the gradually developing permeability to NMDG^+^ and the lack of receptor deactivation after agonist washout (Jiang et al., 2005). This finding indicates that NMDG^+^ cannot substitute for Na^+^ in receptor deactivation (Yan et al., 2008), in contrast to Ca^2+^ (Figure 1B). Under these experimental conditions, a shift also occurred in the reversal potential (Figure 1F), indicating that the kinetics of decay from an outward to an inward current reflects pore dilation. Certainly, NMDG^+^ only partially substituted for inorganic cations as the conducting ion. This phenomenon is indicated by the peak amplitude of the inward current in Figures 1A–C. The time needed for the development of pore dilation was also observed in experiments where NMDG^+^ was substituted for physiological cations during early and sustained agonist application (Figures 1H, I).

When expressed in *Xenopus* oocytes, rP2X4R exhibited a similar biphasic current response, with I_2_ developing slowly over several minutes. The biphasic current was also observed when using NMDG^+^-containing medium. The I_2_ growth occurred simultaneously with a positive shift in reversal potential and an uptake of YO-PRO-1 (Khakh et al., 1999). Other authors also observed pore dilation in rP2X4R expressed in *Xenopus* oocytes that were bathed in a low Ca^2+^ medium (Toulme et al., 2006).

Thus, the P2X4R and P2X7R permeation path is not a single step transition from a small to a large size pore. Instead, the receptor undergoes progressive dilation. Furthermore, the process of rP2X7R dilation was Ca^2+^-independent, in contrast to P2X7R gating, which was allosterically influenced by the presence of Ca^2+^ (Yan et al., 2011). However, rP2X4R pore dilation was blocked by bath Ca^2+^ (Khakh et al., 1999). A model of rP2X7R kinetics was proposed in accordance with these findings, suggesting the coupling of kinetic transitions from the open state to either the closed-desensitized state or to an additional open (dilated) state, which is also the sensitized/facilitated state. The transition to this open sensitized state is kinetically favored over the transition to the desensitized state, leading to the generation of a biphasic current response during the initial agonist application (Yan et al., 2010; Khadra et al., 2013).

## Pore dilation is masked by receptor desensitization

**Figure 2:**
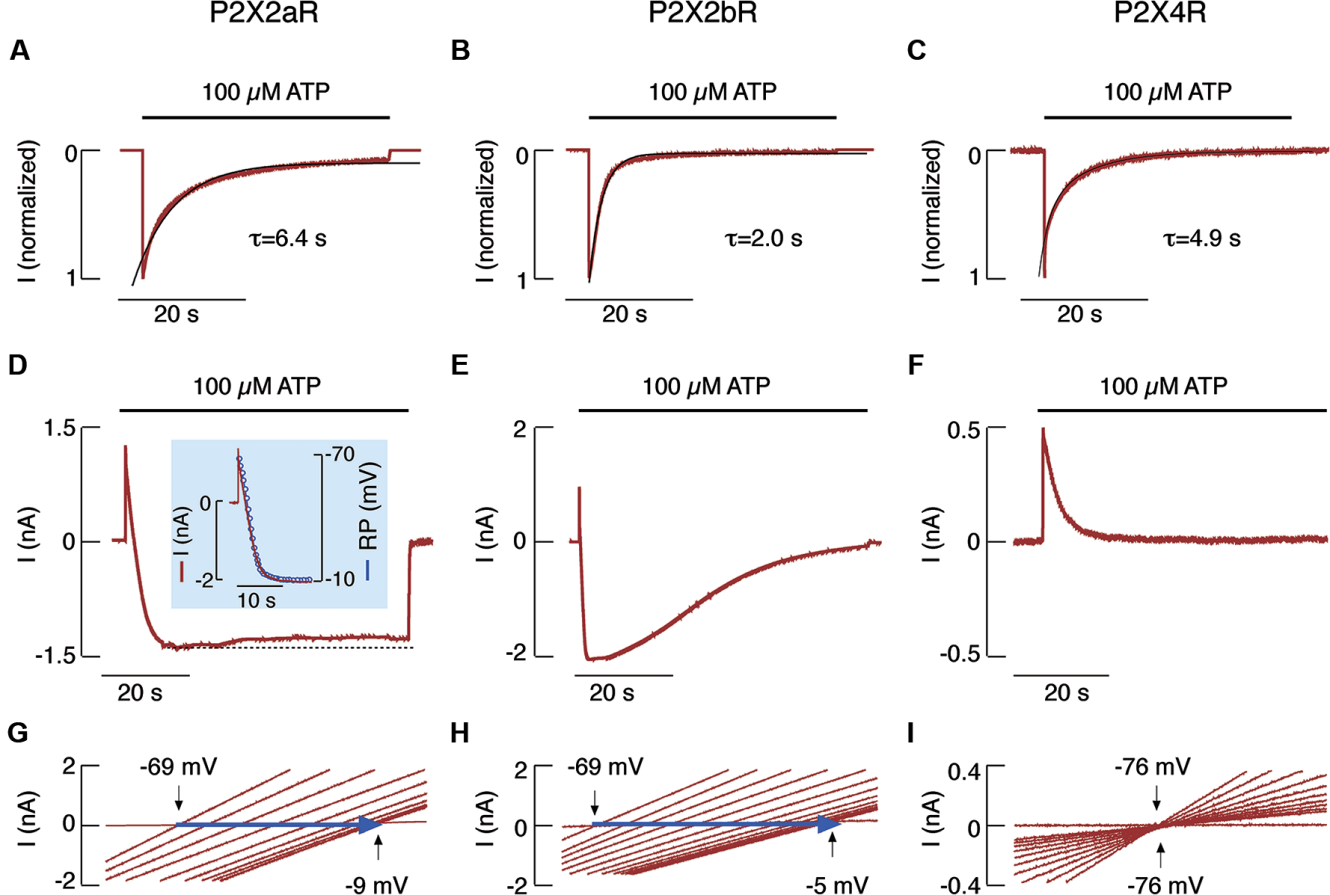
**NMDG^+^ permeability of rP2X2aR, rP2X2bR and rP2X4R expressed in HEK293 cells**. **(A–C)** Patterns of ATP-induced currents in cells bathed in a normal physiological medium. Traces shown were obtained during the first (P2X4R) and third (P2X2aR and P2X2bR) ATP application. Black lines indicate exponential fittings, with *τ_des_* shown below the traces. **(D–I)**. Patterns of ATP-induced current **(D–F)** and reversal potential **(G–I)** in cells that have been continuously bathed in a medium containing only NMDG^+^. (**D**, inset) Temporal correlation between P2X2aR current (red trace) and reversal potential (blue circles). Note the lack of inward current **(F)** and the positive shift in reversal potential **(I)** in cells expressing P2X4R. Derived from Khadra et al. (2012).

When expressed in HEK293 cells, the full size rat receptor (rP2X2aR) and the splice variant missing 69 C-terminal amino acids (rP2X2bR) (Brandle et al., 1997; Koshimizu et al., 1998) each rapidly generated outward currents followed by slowly developing inward currents. Each also exhibited shifts in reversal potential when bathed in NMDG^+^- containing media and uptake of YO-PRO-1 when bathed in physiologically normal buffer. Together, these findings indicate pore dilation in both rP2X2Rs (Virginio et al., 1999b). However, when bathed in a Ca^2+^-containing medium, both forms of rP2X2Rs, as well as rP2X4R, generated desensitizing currents during sustained agonist application; notably, rP2X2bR was desensitized more rapidly than rP2X2aR (Figures 2A–C). The kinetics of pore dilation revealed the splice variant-dependent specificities of this process (Khadra et al., 2012). Cells clamped at −60 mV and stimulated with 100 μM ATP showed an initial rapid outward current, reflecting an efflux of intracellular Na^+^ through the pore. This activity was accompanied by a current decline, which also shifted directionality from outward to inward, reflecting the development of permeability to NMDG^+^ (Figures 2D, E). In contrast, there was no shift from outward to inward current in HEK293 cells expressing rP2X4R (Figure 2F). In cells expressing rP2X2aR and rP2X2bR, but not in those expressing rP2X4R, the substitution of extracellular Na^+^ with NMDG^+^ also resulted in a strong time-dependent shift in reversal potential after the application of voltage-ramp pulses from −80 to +80 mV (Figures 2G–I). Finally, a correlation was found between the development of the NMDG^+^-induced current and the temporal changes in reversal potential (Figure 2D, inset).

Thus, the kinetics of desensitization for rP2X4R, but not for rP2X2aR or rP2X2bR, is identical in cells bathed in physiological and NMDG^+^-containing buffers. Furthermore, rP2X2aR and rP2X2bR, but not rP2X4R, dilate when bathed in NMDG^+^-containing media. Recently, we showed that, in contrast to rP2X2R pore dilation, the removal of bath Ca^2+^ accounts for the difference in rP2X2aR and rP2X2bR desensitization kinetics in cells bathed in physiological and NMDG^+^-containing media (Coddou et al., 2012). Both P2X2R homomers and rP2X2/X5R heteromers expressed in HEK293 cells dilate during sustained receptor activation (Compan et al., 2012). Channel clustering is not obligatory for P2X2R pore dilation, which most likely reflects permissive motions at the interface between the first and second transmembrane domains of neighboring subunits (Khakh and Egan, 2005). Finally, an experimentally supported mathematical model provides a rationale for the lack of sustained current growth in dilating rP2X2Rs by showing that dilated receptors also desensitize in the presence of Ca^2+^ (Khadra et al., 2012). The Ca^2+^-dependent transition from dilated to desensitized states does not occur in rP2X7R-expressing cells. This receptor only shows Ca^2+^-independent desensitization, resulting in a biphasic current response (Khadra et al., 2013).

## Pannexin-1 and p2xr signaling

A comparative study of NMDG^+^ and YO-PRO-1 uptake with wild type and mutant rP2X7Rs raised the possibility that these two molecules do not enter the cell by the same permeation pathway (Jiang et al., 2005). The search for a pathway accounting for YO-PRO-1 uptake led to the hypothesis that pannexin-1 (Panx1) could mediate pore formation and interleukin-1β release by rP2X7R (Pelegrin and Surprenant, 2006). Consistent with this hypothesis, Panx1 was found to co-immunoprecipitate with rP2X7R (Pelegrin and Surprenant, 2006; Li et al., 2011b) and other rP2XRs (Li et al., 2011b) when co-expressed in HEK293 cells.

However, several lines of evidence suggest that pore dilation is an intrinsic property of P2XR channels, independent of Panx1 expression and function. First, C6 astroglioma cells lack endogenously expressed Panx1, but the rP2X7R expressed in these cells dilates (Yan et al., 2008). Second, rP2X2R and rP2X7R dilation was not affected by overexpression of Panx1 or by blockading this channel with carbenoxolone (Chaumont and Khakh, 2008; Yan et al., 2008). Third, dilation of native mP2X4R bathed in a low Ca^2+^ medium was unaffected by carbenoxolone (Bernier et al., 2012). Fourth, RNAi targeting Panx1 did not affect native mP2X7R pore dilation (Alberto et al., 2013). Fifth, recent simultaneous measurement of membrane currents, fluorescent dye uptake, and permeation pathway sizing revealed that the dilated channel of rP2X7R allows the passage of molecules as large as 1.4 nm (Browne et al., 2013). Sixth, recent literature suggests that Panx1 may contribute to ATP release (Locovei et al., 2006; Huang et al., 2007; Iglesias et al., 2009; Li et al., 2011a); the role of Panx1 in ATP release but not in P2X7R pore dilation was further supported by experiments using Panx1 knockout bone marrow-derived macrophages (Qu et al., 2011).

## The role of cytosolic domains in P2XR gating

The initial observation of the role of the C-terminus of rP2X7R in YO-PRO-1 uptake was reported by Surprenant et al. (1996). hP2X7R expressed in *Xenopus* oocytes also exhibited C-terminus-dependent gating properties (Becker et al., 2008), but there were some differences in responses of rP2X7R and hP2X7R when expressed in HEK293 cells, probably reflecting the receptor-specific C-terminal domain structure (Rassendren et al., 1997). Studies comparing the wild type and C-terminal truncation forms of rP2X7R expressed in HEK293 cells and *Xenopus* oocytes further indicated the importance of the C-terminus for receptor dilation (Smart et al., 2003). Rat, but not mouse, P2X2aR showed pore dilation when expressed in *Xenopus* oocytes, thus suggesting the role of specific cytosolic domains as determinants of permeation in a state-specific manner (Eickhorst et al., 2002). The removal of a cysteine-rich segment of the intracellular juxta-membrane region of rP2X7R was reported to cause the loss of NMDG^+^ permeability without affecting YO-PRO-1 uptake (Jiang et al., 2005). This discrepancy can be explained by the instantaneous opening of the mutant receptor into the dilated state (Yan et al., 2008). The N-terminal T15 mutants also opened instantaneously into the dilated state in a protein kinase C-independent manner (Yan et al., 2008, 2010), suggesting that N-terminal contributes to the control of transition from open to dilated state.

## Future directions

Although the crystal structures of the zP2X4.1R channel in both the ATP-bound open state and the apo-closed state have been solved (Kawate et al., 2009; Hattori and Gouaux, 2012), the structure of the dilated state is missing, presumably due in part to the lack of N- and C-termini in crystallization studies. The structural correlate of the P2X7R sensitization/facilitation state (Roger et al., 2008; Yan et al., 2010) is also missing. The dilated state is strongly ATP sensitized (Yan et al., 2010), which could imply structural changes in the ATP binding site and/or different orientations of bound ATP (Jiang et al., 2011). Pore dilation was not observed in single channel recording (Tatham and Lindau, 1990; Ding and Sachs, 1999; Riedel et al., 2007a,b) and the open-1 state was estimated to be 0.85 nm (Riedel et al., 2007b). The reason for this discrepancy is not clear. Mutagenesis studies have identified specific regions and residues of P2XRs that influence pore dilation (Khakh and Egan, 2005; Yan et al., 2008; Sun et al., 2013), but further crystallization and mutagenesis studies are needed to better understand the transition from open to dilated states. It is also unclear which physiologically relevant metabolites from the cellular microenvironment permeate through the dilated P2XR pore, or whether there are any allosteric up- or down-regulatory mechanisms that could convert the receptor to the dilated or naïve closed states. P2X7R exhibits a significant number of gene polymorphisms with strong pathophysiological implications in hP2XRs (Wesselius et al., 2011); further investigations should clarify whether the loss- or gain-of-function variants shows pore dilation. The development of pharmacological tools for altering the transition of P2XR pores from open to dilated states could help in dissecting the physiological significance of the two open states of P2XRs.

## Conflict of interest statement

The authors declare that the research was conducted in the absence of any commercial or financial relationships that could be construed as a potential conflict of interest.
